# Immunomodulatory properties of quercetin-3-O-α-L-rhamnopyranoside from *Rapanea melanophloeos* against influenza a virus

**DOI:** 10.1186/s12906-018-2246-1

**Published:** 2018-06-15

**Authors:** Parvaneh Mehrbod, Muna Ali Abdalla, Fatemeh Fotouhi, Masoumeh Heidarzadeh, Abimbola O. Aro, Jacobus N. Eloff, Lyndy J. McGaw, Folorunso O. Fasina

**Affiliations:** 1 Department of Veterinary Tropical Diseases, University of Pretoria, Pretoria, South Africa; 2 Influenza and Other Respiratory Viruses Department, Pasteur Institute of IRAN, Tehran, Iran; 3 Phytomedicine Programme, Department of Paraclinical Sciences, University of Pretoria, Pretoria, South Africa; 4 Department of Food Science and Technology, Faculty of Agriculture, University of Khartoum, 13314 Khartoum North, Sudan; 5 ECTAD, Food and Agriculture Organization of the United Nations (FAO), House H Sida, Ada Estate, P.O.Box 2, Dar es Salaam, Tanzania

**Keywords:** *Rapanea melanophloeos*, Influenza a virus, Quercetin-3-O-α-L-rhamnopyranoside, Cytokine

## Abstract

**Background:**

Influenza infection is a major public health threat. The role of influenza A virus-induced inflammatory response in severe cases of this disease is widely recognized. Drug resistance and side effects of chemical treatments have been observed, resulting in increased interest in alternative use of herbal medications for prophylaxis against this infection.

The South African medicinal plant, *Rapanea melanophloeos* (RM) (L.) Mez of the family Myrsinaceae was selected owing to its traditional use for the treatment of several diseases such as respiratory ailments and also previous preliminary studies of anti-influenza activity of its methanolic extract. The aim of this study was to investigate the immunomodulatory properties of a glycoside flavone isolated from RM against influenza A virus.

**Methods:**

The non-cytotoxic concentration of the quercetin-3-O-α-L-rhamnopyranoside (Q3R) was determined by MTT assay and tested for activity against influenza A virus (IAV) in simultaneous, pre-penetration and post-penetration combination treatments over 1 h incubation on MDCK cells. The virus titer and viral load targeting NP and M2 viral genes were determined using HA and qPCR, respectively. TNF-α and IL-27 as pro- and anti-inflammatory cytokines were measured at RNA and protein levels by qPCR and ELISA, respectively.

**Results:**

Quercetin-3-O-α-L-rhamnopyranoside at 150 μg/ml decreased the viral titer by 6 logs (*p* < 0.01) in the simultaneous procedure. The NP and M2 genes copy numbers as viral target genes, calculated based on the Ct values and standard formula, significantly decreased in simultaneous treatment (*p* < 0.01). The expression of cytokines was also considerably affected by the compound treatment.

**Conclusions:**

This is the first report of quercetin-3-O-α-L-rhamnopyranoside from RM and its immunomodulatory properties against influenza A virus. Further research will focus on detecting the specific mechanism of virus-host interactions.

**Electronic supplementary material:**

The online version of this article (10.1186/s12906-018-2246-1) contains supplementary material, which is available to authorized users.

## Background

Influenza A virus (IAV) (family Orthomyxoviridae, type *A*) causes severe upper respiratory diseases in humans as well as in different animal species, resulting in considerable morbidity and mortality [1, 2]. The acquisition of resistance to chemical drugs such as M2 and NA inhibitors, [3] mainly due to antigenic shifts and drifts, is a potential problem [2, 3]. This issue has led to the caution from the Centers for Disease Control and Prevention (CDC) over the continued use of these drugs [4].

Amongst many considerations, influenza infection can induce a cytokine storm or ‘hypercytokinemia’, a situation of overproduction of immune cells and their activating compounds (cytokines) which may become potentially fatal, as a result of a positive feedback loop between cytokines and immune cells [5]. Thus treatments targeting inflammatory responses are pivotal.

The use of herbal medicine has been accepted in many countries, including regions with improved healthcare systems [6, 7]. Medicinal plants are becoming increasingly popular in modern society as complementary therapies and as preventive medicine [8–10]. Studies to determine the chemical profile and composition of medicinal plants have revealed the complexity and variety of compounds all contributing to the various uses of plants in treating numerous diseases including life-threatening bacterial and viral diseases, and cancers [11].

Different medicinal plants have been evaluated for antiviral activity against different viruses such as picornaviruses, herpes simplex viruses types 1 and 2 (HSV-1 and 2), influenza virus type A (Inf A) and human immunodeficiency virus type 1 (HIV-1) [12–14]. Although medicinal plants have been exploited by traditional societies against certain diseases, the safety of the crude extract must be evaluated as some phytochemicals may exist at toxic levels in crude extracts [15]. The bioactivity may also be suboptimal because maximum activity requires certain combinations of phytochemicals [16].

Phytochemical screening of *Rapanea melanophloeos* (RM), a medicinal plant used by Zulu traditional healers, showed the presence of tannins, terpenoids, alkaloids, saponins, cardiac glycosides, flavonoids and phlobatannins [17]. This plant has been used against fever, cough, chest disease, night sweats etc. As an alternative approach to the common antivirals, the methanolic crude extract of RM had antiviral efficacy in our preliminary studies [18] and is worthy of further study. The objective of the current study therefore was to investigate the mechanism of anti-influenza activity of the glycoside flavone named quercetin-3-O-α-L-rhamnopyranoside (Q3R) isolated from RM with regard to its immunomodulatory properties.

## Methods

### Plant material, extraction and isolation of quercetin-3-O-α-L-rhamnopyranoside

The plant was collected from the Pretoria National Botanical Garden (NBG), South Africa in the summer months. One of the authors (LJM) identified the plant material and a voucher specimen was deposited in the HGWJ Schweickerdt Herbarium (PRU), University of Pretoria, South Africa. The plant material and the crude extract were prepared as reported in Mehrbod et al., 2018 [18]. The methanol crude extract of *R. melanophloeos* showed remarkable antiviral activity against IAV. The average of 7.4 log HA decrements were observed in all types of combined treatments of *R. melanophloeos* [18]. The extract was evaporated to dryness and subjected to silica gel column chromatography chloroform/methanol (gradient 0 to 100% methanol) to afford four fractions. Fraction FIII was purified twice on Sephadex LH-20 column chromatography using MeOH to obtain quercetin-3-O-α-L-rhamnopyranoside in pure form.

### Structure identification of quercetin-3-O-α-L-rhamnopyranoside

Quercetin-3-O-α-L-rhamnopyranoside was characterized by means of nuclear magnetic resonance (NMR) (1D and 2D) spectroscopic and mass spectrometry data. 1H NMR and 2D NMR experiments data were acquired on a 400 MHz NMR spectrometer (Bruker Avance III 400 MHz). Compound detection was performed using a Waters® Synapt G2 high definition mass spectrometry (HDMS) system (Waters Inc., Milford, Massachusetts, USA). The system comprises of a Waters Acquity Ultra Performance Liquid Chromatography (UPLC®) system hyphenated to a quadrupole-time-of-flight (QTOF) instrument. The system was operated with MassLynxTM (version 4.1) software (Waters Inc., Milford, Massachusetts, USA) for data acquisition and processing. An internal lock mass control standard, 2 pg/μL solution leucine enkephalin (m/z 555.2693), was directly infused into the source through a secondary orthogonal electrospray ionisation (ESI) probe allowing intermittent sampling. The internal control was used to compensate for instrumental drift, ensuring good mass accuracy.

### Cell culture and influenza virus propagation

Madin Darby Canine Kidney (MDCK) cells (CCL-34™) obtained from Pasteur Institute of Iran, Department of Influenza and Other Respiratory Viruses, were grown in Dulbecco’s Modified Eagle’s Medium (DMEM) (Gibco USA), supplemented with 10% Fetal Bovine Serum (FBS) (Gibco USA) and 1% Pen/Strep (Gibco USA) at 37 °C in a humidified 5% CO_2_ incubator. The influenza virus vaccine strain, A/Puerto Rico/8/1934 (H1N1) (ATCC VR-1469™) obtained from Influenza Department, Pasteur Institute of Iran was propagated in MDCK cells. DMEM supplemented with 1 μg/ml of Trypsin-TPCK (Tosylamide Phenylethyl Chloromethyl Keton-treated Trypsin) (Sigma, USA) without FBS was used as maintenance medium during antiviral experiments. The virus infectivity dose was measured using cell culture infectious dose 50 (CCID_50_) in combination with the hemagglutination assay [19, 20].

### Cytotoxicity of quercetin-3-O-α-L-rhamnopyranoside

The cytotoxicity of Q3R against MDCK cells was determined by the MTT [3-(4,5-dimethylthiazol-2-yl)-2,5-diphenyltetrazolium bromide] assay [21, 22]. The cells were seeded in 96-well microtitre plates (Nunc, Denmark) (3 × 10^4^ cell/well) and incubated at 37 °C in a humidified 5% CO_2_ incubator overnight. Then, 2-fold serial dilutions of Q3R in DMEM (100 μl) were added to the cells in triplicate and incubated for more 48 h. Doxorubicin hydrochloride (Pfizer) was used as a positive control. The cells without treatment and cells exposed to dimethylsulfoxide (DMSO) with maximum 0.5% concentration were used as negative and vehicle controls, respectively. After incubation, the colorimetric MTT viability assay was carried out as described before. The cell survival rate was calculated using the following formula: (mean Optical Density (OD) of treated cells/mean OD of control cells) × 100. The 50% cytotoxic concentration (CC_50_) was defined as the concentration which causes visible morphological changes in 50% of the cells based on the observation under inverted microscope with respect to the control cells. A non-cytotoxic concentration (NCTC) was used for antiviral assays.

### Dose-dependent response assay

The H1N1 virus (100 CCID_50_/0.1 ml) 0.5 multiplicity of infection (MOI) was exposed to 70–80% confluent MDCK cells in combination with different dilutions of the compound (2 wells for each dilution) from 200 to 6.25 μg/ml (100 μl/ well) for 1 h at 37 °C (100 μl/0.5 MOI) in 96-well flat-bottom micro-plate (Nunc, Denmark). Following the incubation time, the supernatants were removed and TPCK-containing medium was added to each well. The plate was incubated at CO_2_-incubator for 48 h. The viability of the infected and non-infected cells was evaluated by MTT assay as mentioned before. The virus titration was carried out using the Hemagglutination Assay (HA). Double serial dilutions of the culture media were added to U-bottom 96-well microplates. Washed chicken red blood cells (cRBCs) (1% volume in PBS) were added to each well. The assay was carried out as described previously [23] and modified [22].

### Antiviral activity of the compound

In brief, MDCK cells were treated with compound (NCTC) for 1 h, then were washed before viral infection (100 CCID_50_/0.1 ml) for 1 h (pre-penetration treatment), compound and virus were mixed for 30 min at room temperature and added to the cells together for 1 h infection period (co-penetration treatment), or compound was added for 1 h right after the infection period (post- penetration treatment). Following 1 h incubation, unabsorbed viruses were washed and TPCK-containing medium (1 μg/ml) was added. Amantadine hydrochloride and oseltamivir carboxylate (Sigma, Saint Louis, Missouri, USA) were tested in parallel as control antiviral groups. The cells with media only served as negative controls. Following 48 h incubation at 37 °C, viabilities of the cells were evaluated by MTT viability assay as described earlier (Merhbode et al., 2018). Concurrently, the cell supernatants were exposed to HA test to determine the virus titer.

### RT-qPCR analysis of the selected genes

For this step, MDCK cells were treated as before. The supernatants and cells were harvested for RNA extraction. The supernatants were used to extract the extracellular viral RNA by High Pure Viral Nucleic Acid Kit (Roche, Germany) according to the manufacturer’s protocol. For intracellular RNA, the collected cells were centrifuged to make a pellet. Then, a High Pure RNA Isolation Kit was used to extract the total RNA according to the kit instruction (Roche, Germany). The RNA samples were stored aliquoted at − 80 °C.

All RNA samples were subjected to cDNA synthesis using a Transcriptor First Strand cDNA Synthesis kit (Roche, Germany) including 5X Transcriptor Reverse Transcriptase Reaction buffer, Random Hexamer primers, Protector RNase Inhibitor, dNTP mix and Transcriptor Reverse Transcriptase in a final volume of 20 μl. The mix was incubated at 25 °C for 10 min for primer annealing followed by 55 °C for 30 min for reverse transcription and inactivated at 85 °C for 5 min. The synthesized cDNAs were stored at − 20 °C for further usage. The concentration of the cDNA templates was measured using a Picodrop Spectrophotometer system (Alpha, Biotech, UK). Virus-inoculated and mock-infected samples were considered as positive and negative controls, respectively.

The primers for the selected viral genes were designed by First Base Co. Malaysia. The primers of the selected cytokines and housekeeping genes were designed by Next Gene Co. Malaysia. All primers were synthesized by Inqaba Biotech Co. South Africa. The target genes consist of two viral genes (NP and M2), two cytokines (TNF-α and IL-27), and two housekeeping genes (Gus-B and Act-B). Table 1 shows the specification of these primers.

**Table 1:** The primers specification for amplification of the targeted genes

Gene name	Primer sequence (5′ to 3′)	Accession number	Position	Size (bp)
PR-NP-F	TCAGTGATTATGAGGGACGGrUTGAT/3Sp	CY148246.1	179–198	97
PR-NP-R	TTCTTCCAGGTATTTATTTCTCCTrUTCGTT/3Sp		253–276	
PR-M2-F	GCAGTTAAACTGTATAGGAAGCTrCAAGA/3Sp	CY148244.1	311–333	69
PR-M2-R	CACCAGCAGAATAACTGAGTGrAGATTC/3Sp		360–380	
TNF-α-F	ATCAATCTGCCTAACTATCT	NM_001003244.4	634–653	168
TNF-α-R	CTGAGCCCTTAATTCTCT		785–802	
IL-27-F	GCTGTTCTCAGAGGTTCGG	XM_844736.3	258–276	75
IL-27-R	CAGGAGGTCCAGGCTTACT		315–333	
GusB-F	TGCTCCTCTACACCACACCTAC	NM_001003191.1	532–553	80
GusB-R	CCACCAGCCCAGTGTCTTG		594–612	
ACTB-F	CAGGAGTACGACGAGTCCG	NM_001195845.1	1209–1227	87
ACTB-R	CAAGAAAGGGTGTAACGCAACT		1275–1296	

Real-time PCR reactions were performed using Light Cycler FastStart DNAMaster SYBR Green I (Roche, Germany) with related primers using Corbett Rotor-Gene Q 6000 (Corbett Research, Australia) in a total volume of 20 μl. All PCR materials were mixed and prepared in 0.2 ml PCR tubes in the dark. Thermal cycling program was performed using three-step cycling protocol according to the manufacturer’s instructions. All the PCR reactions were performed in duplicate accompanied by a non-template control (NTC).

For the absolute quantification of viral genes, the copy number in each treatment was calculated using the following formula [24]:

Number of copies/μl = [6.02 × 10^23^ (molecules/mole) × DNA concentrations (g/μl)]/ [Number of bases pairs × 660 Da].

The number 6.02 × 10^23^ (molecules/mole) is Avogadro’s number and 660 Da is the average weight of a single base pair.

The efficiency for the gene was calculated by drawing a standard curve from a 10-fold serial dilution of one of the samples with high amounts of the target gene. The resultant standard curves of the gene Ct values versus the gene copy numbers was used to calculate the absolute quantification of the genes copy numbers in the treatments.

For the relative expression analysis of cytokines genes, the ΔΔCt method was used to analyze the data. In this approach, all the quantified Ct values were standardized by the reaction efficacy and the related reference gene expression (average of the Ct values of the two housekeeping genes).

### Cytokine protein quantification with ELISA

MDCK cells were treated as stated above. Untreated MDCKs were considered as the negative control. The cell-free supernatants were harvested following 48 h incubation and stored at − 80 °C for the cytokine analysis. All the samples were tested in duplicate. The expression level of TNF-α and IL-27 following treatments was evaluated by quantitative sandwich Picokine ELISA kits (Boster Biological Technology, CA, USA) according to the manufacturer’s instructions. The optical density of the wells was measured using microplate reader (Anthos 2020, version 2.0.5) at 450 nm wavelength. The density of yellow color is proportional to the cytokine amount in the sample. The concentrations of the cytokines were calculated according to the corresponding reaction standard formula.

### Statistical analysis

The data expressed as mean ± SD was analyzed by one-way analysis of variance (ANOVA (SPSS 18.0) with the Tukey post-hoc test. Sample values with *p* ≤ 0.05 and *p* ≤ 0.01 were considered statistically significant and highly significant, respectively.

## Results

### Structure characterization of quercetin-3-O-α-L-rhamnopyranoside

**Fig. 1:**
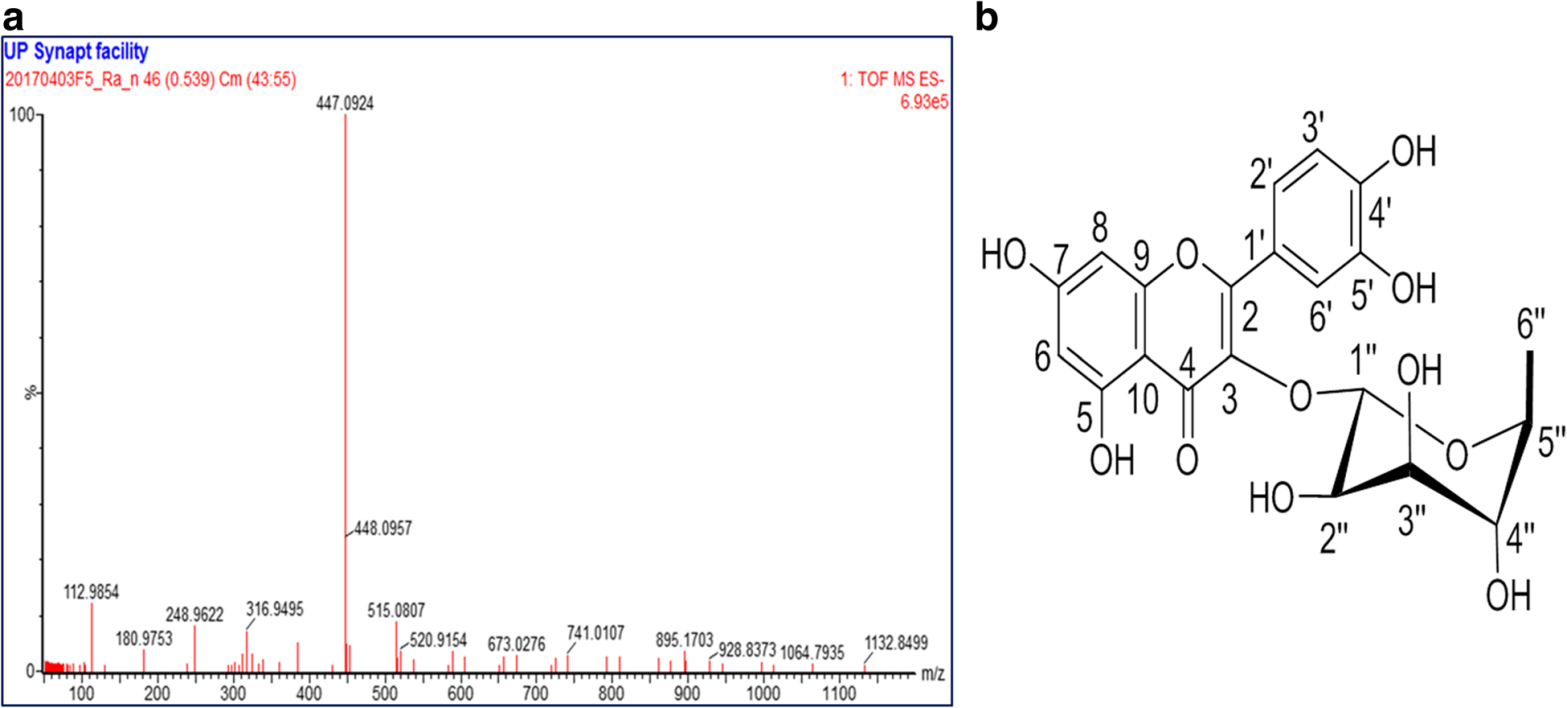
ESI/MS spectrum of quercetin-3-O-α-L-rhamnopyranoside (**a**). Structure of the quercetin-3-O-α-L-rhamnopyranoside (**b**)

Quercetin-3-O-α-L-rhamnopyranoside was isolated as a yellow powder, which gave a strongly UV absorbing band on TLC at 254 nm and turned to yellow upon exposure to the vanillin-sulphuric acid reagent. The molecular formula of the isolated compound was determined to be C_21_H_20_O_11_ as derived from its negative mode ESI-MS (m/z 447.0900 [M-H]−) as shown in Fig. 1a. The ^1^H NMR and ^13^C NMR data are presented in Table 2. ^1^H, ^13^C, H:H COSY, HMBC and HSQC spectra are presented in the Additional files 1, 2, 3, 4 and 5. A search in the Dictionary of Natural Products [25] and comparing the spectroscopic and MS data with the literature confirmed the structure as quercetin-3-O-α-L-rhamnopyranoside (Fig. 1b).

**Table 2:** ^1^H NMR and ^13^C NMR data of compound 1 (Q3R) (in DMSO-*d*_*6*_)

Position	Compound 1 (Q3R)	
	H	C
2	–	157.7
3	–	134.8
4	–	178.3
5	–	162.1
6	6.27, d, *J* = 2.1 Hz	98.5
7	–	164.7
8	6.49, *J* = 2.1 Hz	93.9
9	–	157.3
10	–	104.6
1’	–	122.1
2’	7.41, dd, *J* = 2.2, 8.3 Hz	121.8
3’	6.99, d, *J* = 8.3 Hz	115.3
4’	–	148.2
5’	–	144.8
6’	7.52, d, *J* = 2.0 Hz	115.9
1’’	5.53, brd s	101.7
2’’	4.24, m	70.4
3’’	3.76, dd, *J* = 3.8, 9.2 Hz	71.0
4’’	3.38, d, *J* = 9.4 Hz	72.0
5’’	3.44, dd, *J* = 6.1, 9.5 Hz	70.4
6’’	0.93, d, *J* = 6.1 Hz	16.7

### Cytotoxicity results

Based on MTT results for the cytotoxicity assay, the CC_50_ and NCTC of the compound were obtained at 200 and 150 μg/ml, respectively. Amantadine hydrochloride and oseltamivir carboxylate CC_50_ values in MDCK cells were calculated as 197 μg/ml and 788 μg/ml, respectively. Concentrations of 98.5 and 394 μg/ ml were used as NCTC of amantadine and oseltamivir, respectively.

### Dose-dependent antiviral response

Different concentrations of the Q3R (200, 150, 100, 50, 25, 12.5 and 6.25 μg/ml) were tested for cell viability and antiviral activity by MTT and HA assays, respectively.

**Fig. 2:**
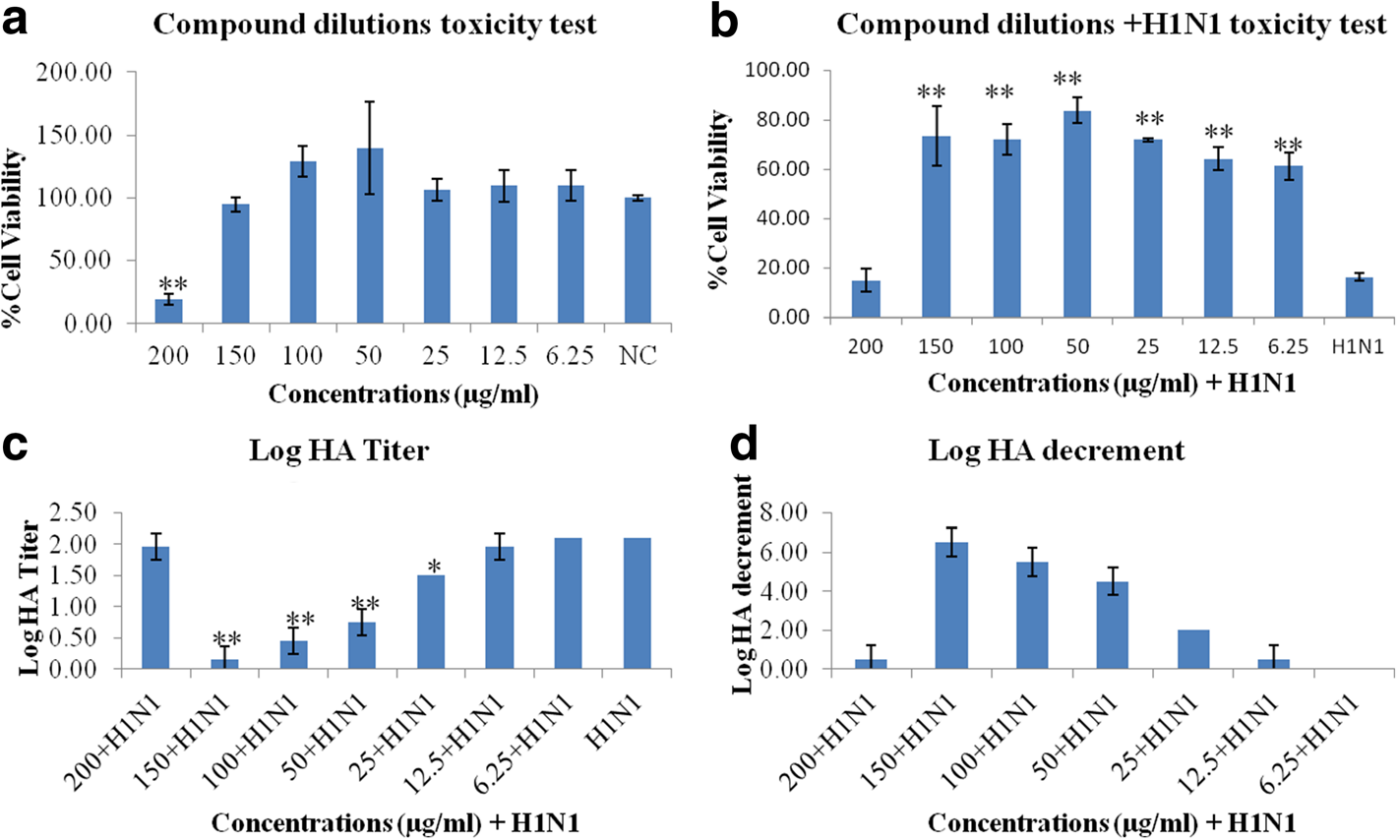
Dose-dependent antiviral response. Compound dilutions toxicity (**a**), Compound dilutions + H1N1 toxicity (**b**), Log HA Titer (**c**) and Log HA decrement (**d**). * & ** show the significant and highly significant differences compared to control, respectively

None of the concentrations showed significant differences in cell viability compared to the negative control, except for 200 μg/ml (*P* ≤ 0.01). These concentrations in combination treatments with H1N1 showed increased cell viability compared to H1N1 alone (P ≤ 0.01), except for 200 μg/ml. The HA titers showed dose-dependent responses with the compound concentrations. However, the 200 μg/ml concentration could not decrease the HA titer significantly. The EC_50_ and EC_90_ of the compound were calculated at 25 and 100 μg/ml, respectively. The relative safety of the compound was confirmed by calculating the selectivity index (SI) which is calculated by dividing the CC_50_ by the EC_50_. The SI was 8 which is considered a good value as values higher than 3 indicate potentially safe antiviral activity [26].The highest Log HA decrement was observed for 150 μg/ml concentration. This concentration was chosen for the molecular and biochemical assays. Figure 2 shows the results of the MTT (A,B) and HA (C,D) assays.

### Anti-influenza activity of quercetin-3-O-α-L-rhamnopyranoside

The amount of virus used was based on infected target cells of 0.5 multiplicity of infection (MOI) [26]. During antiviral evaluations, media supplemented with FBS was removed and the cells were washed with PBS and then treated as mentioned above for experimental procedures.

Based on HA titration, the inhibitory effect of the compound on viral adsorption to the cell surface in different treatments was demonstrated by a significant reduction in the HA titer unit especially in the co-penetration treatment (*P* ≤ 0.01) which decreased the viral titer to zero. But in the pre- and post-penetration procedures the viral titer decreased by approximately 1 log at 48 h.

**Fig. 3:**
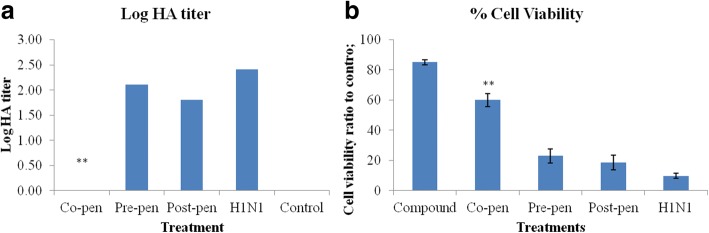
The effects of the compound (150 μg/ml) on HA titer (**a**) and cell viability (**b**) in different combination treatments. ** shows the highly significant difference compared to control

Increased optical density correlating with increased cell viability in the combined treatments of compound NCTC and H1N1 compared to H1N1 alone was markedly significant in the co-penetration treatment (*P* ≤ 0.01) but not in the pre- and post- treatments. The significant increase in cell viabilities as compared to H1N1 infection demonstrated the protective effect of the compound on the cell viability against viral cytopathic effects. Figure 3 illustrates the HA (A) and MTT (B) assays results. Amantadine and oseltamivir as control antiviral drugs were tested in parallel.

### Copy number and expression changes of the selected genes

#### Absolute quantification

**Fig. 4:**
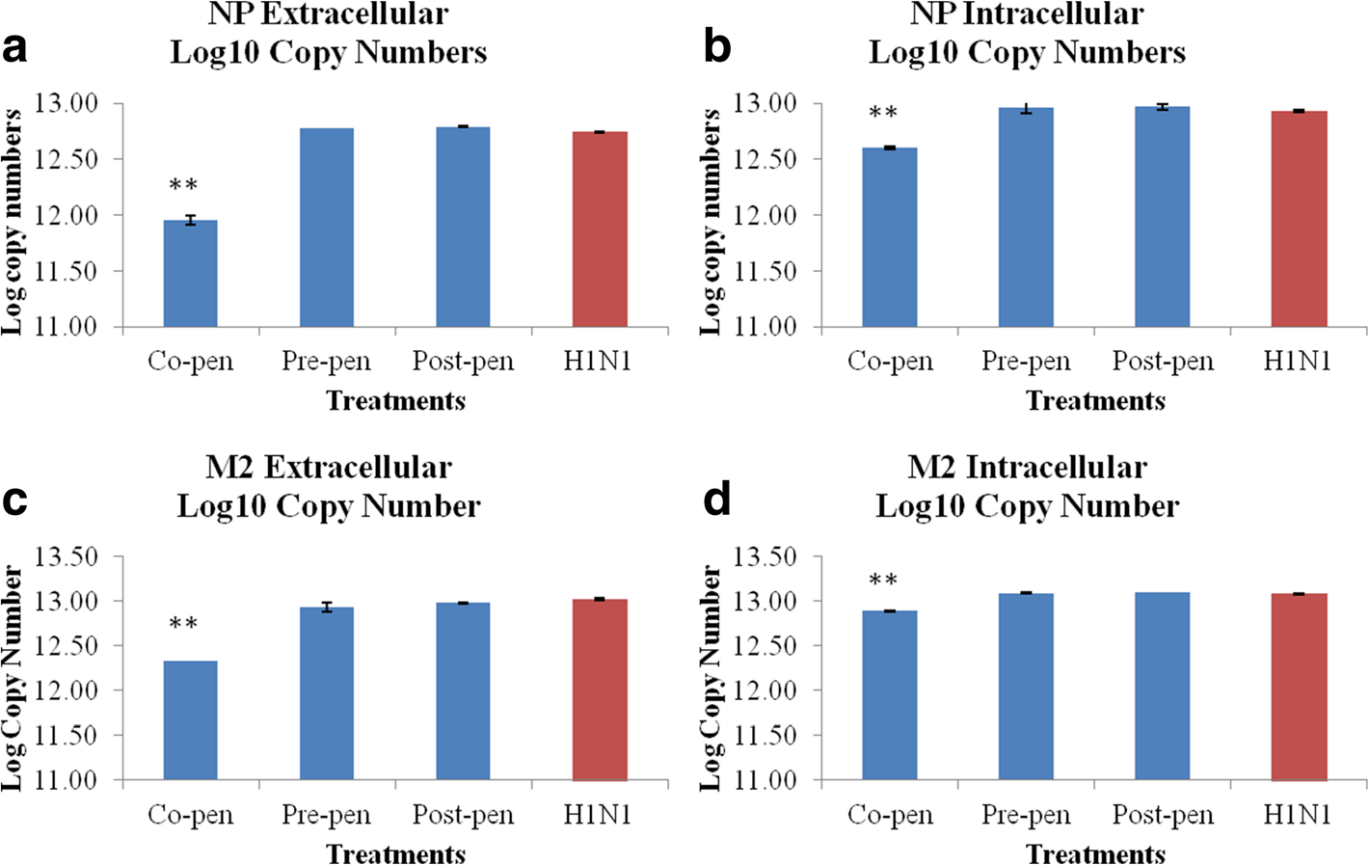
The charts show the NP (**a**, **b**) and M2 (**c**, **d**) genes extracellular and intracellular Log_10_ copy numbers in different combination treatments as compared to H1N1-inoculated sample. **: indicates the highly significant differences with the infected positive control (*p* < 0.01)

The log_10_ copy numbers for the NP and M2 genes standard dilutions were calculated based on the concentrations of the templates and related formula. The standard amplification curves for extra- and intra-cellular NP and M2 genes were generated by plotting cycle threshold values (Ct) against input cDNA log_10_ copy numbers alongside a non-template control (NTC). The viral genes log_10_ copy numbers after different combination treatments of the compound (150 μg/ml) and H1N1 (100CCID_50_/100 μl) were calculated based on the Ct values and the related standard formula obtained from the standard curve. The extracellular and intracellular influenza virus NP and M2 genes copy numbers in different treatments with the compound were calculated. Data are shown in Fig. 4. The data showed the highly significant decrement (*p* < 0.01) in H1N1 log_10_ copy numbers in co-penetration treatments in extracellular samples (A,C) but not significant effect was observed in pre- and post-penetration treatments. There were also significant decrements in H1N1 log_10_ copy numbers in co-penetration treatments in intracellular samples (B,D) (*p* < 0.01).

#### Relative expression analysis

**Fig. 5:**
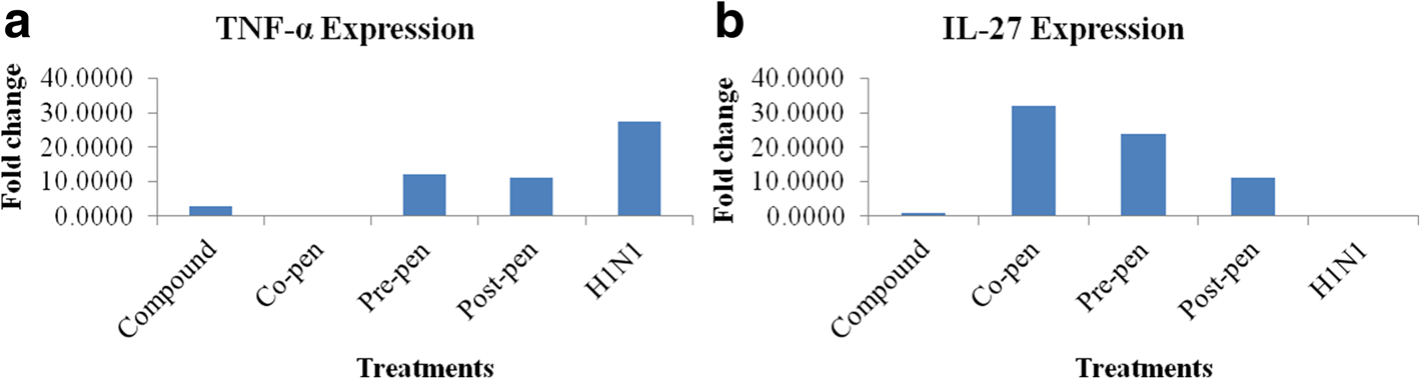
Relative expression analysis (ΔΔCq) of the cytokines TNF-α (**a**) and IL-27 (**b**) compared to the positive control

In this approach, the Ct values of target cytokines were standardized by the reaction efficacy and the related reference genes expression (average of the Ct values of the two housekeeping genes). The relative expression analysis of the cytokine genes were calculated as fold change compared to the negative control. Data are shown in Fig. 5. As can be seen in the Figure, H1N1 inoculation increased TNF-α expression to 27.38 fold but in the co-penetration procedure, the compound treatment decreased this cytokine expression to 0.02 fold (5A). With regard to IL-27, H1N1 decreased this cytokine to 0.0003 fold while in the co-penetration treatment it increased to 31.83 fold (5B).

### Analysis of the cytokines with ELISA

The TNF-α and IL-27 cytokine protein levels in supernatants of MDCK cell culture at 48 h after exposure were calculated according to the reaction standard formula. Data are shown in Table 3. Regarding TNF-α concentration, virus inoculation caused a high level of this pro-inflammatory cytokine while in all combination treatments this protein showed decrements especially in the co-penetration treatment (*p* < 0.01) which highlighted − 83.687% changes to virus sample. Regarding IL-27 concentration, all combination treatments increased IL-27 protein level highly significantly (*p* < 0.01) compared to virus inoculation which highlighted 135.495, 101.802 and 120.901% increases to the virus sample.

**Table 3:** TNF-α and IL-27 proteins concentrations in MDCK culture supernatants (pg/ml) at 48 h treatment

Treatment	TNF-α concentration (pg/ml) (mean ± SD)	TNF-α % change to virus sample	IL-27 concentration (pg/ml) (mean ± SD)	IL-27% change to virus sample
Co-pen	59.36 **±** 0.003^**^	−83.687	2178.33 ± 0.001^**^	+ 135.495
Pre-pen	200.5 **±** 0.007	−44.904	1866.67 ± 0.004^**^	+ 101.802
Post-pen	196.2 **±** 0.010^*^	−46.090	2043.33 ± 0.005^**^	+ 120.901
H1N1	**363.9 ± 0.011**		**925.00 ± 0.006**	

Concentrations of TNF-α and IL-27 and percentages of changes compared to H1N1, as determined by ELISA, are expressed as pg/ml (*N* = 2) for 48 h incubation time

* & **: significantly (*P* < 0.05) and highly significantly (*P* < 0.01) different from H1N1-inoculated sample

## Discussion

Anecdotal evidence supports the traditional use of *Rapanea melanophloeos* for the treatment of several respiratory ailments, and in our previous research we studied the methanolic extract this plant and showed its efficacy against influenza A virus [18]. In this study, the interaction between Q3R, a glycoside flavone isolated from *Rapanea melanophloeos,* and influenza virus A/PR/8/34 was evaluated in vitro. The compound was not toxic on MDCK cells up to 200 μg/ml concentration. Quercetin caused a dose-response reduction in the infectivity of the virus. Dose-response assay proved that 150 μg/ml of the compound was the most effective in significantly reducing the virus titre. This concentration had the highest efficiency in the co-penetration treatment.

The pathogenesis of influenza virus is a combination of the host and virus factors. The virus particle facilitates replication of the virus inside the target cell and also deceives the host immune system. It has been reported that the fatal consequence of influenza is eminently associated with a massive viral load along with high cytokine deregulation, which causes a cytokine storm or hypercytokinemia [27], of both pro- and anti-inflammatory cytokines. Hence, the innate immune system can affect the clinical manifestation and fatality following influenza virus infection [28].

Natural products have become recognised as an excellent source of extracts or compounds useful in controlling viral infection [29]. Flavonoids are plant-derived polyphenolic compounds with many potential health benefits. Different types of flavonoids have been identified as antiviral agents [30–33]. The potent antiviral effect of flavonoids against influenza virus infection [34–36] and immunomodulatory effects of flavonoids in different viral infections [37–39] have been reported. Quercetin from the flavonoid group of plant compounds has been studied in small clinical trials [40]. There are limited studies on immunomodulatory effects of quercetin on influenza infection. One such study indicated the inhibitory activity of quercetin on influenza infection in the early stage of entry [41]. Q3R from *Houttuynia cordata* demonstrated strong anti-influenza A/WS/33 virus activity, reducing the formation of visible CPE, and inhibited virus replication in the initial stage of virus infection [42]. However, according to our knowledge there are no studies on immunomodulatory effect of quercetin during influenza infection. The results of the current study revealed that Q3R has the capacity to directly inhibit virus replication and affect cytokine production.

The targeted viral genes in this study were NP and M2. The NP gene encodes the virus nucleoprotein and M2 channel proteins have a variety of effects on different stages of the virus life cycle. This can be illustrated by viral entry, viral assembly by inhibition of lysosomal activity and autophagosomes [43], and budding of the newly formed virus particles [44]. It was noted that quercetin could decrease both the intracellular and extracellular copy numbers of the genes in the co-penetration treatment which confirms the blockage of the viral particle receptors from penetration inside the cell, thus fewer viral particles propagated inside the cell. No significant effect in pre- and post-penetration treatments verified the inability of the compound to influence the cellular receptors and probably the cellular pathways.

One of the key factors of influenza pathogenesis is modification of cytokine production, which can recruit a variety of innate immune cells [45]. TNF-α and IL-27 were tested from two categories of pro-inflammatory and anti-inflammatory cytokines, respectively. It was seen that quercetin altered the status of cytokine production during the influenza course. One of the affected cytokines was IL-27. This cytokine can increase the production of IL-10 by the antiviral CD4^+^ cytotoxic T lymphocytes (CTLs), which can efficiently modulate excessive immune response injuries [46]. Quercetin could increase the IL-27 production significantly especially in the co-penetration treatment to 135.495% compared to the H1N1 positive control. Moreover, it was able to significantly decrease the TNF-α production to − 83.687% in the co-penetration treatment. Various influenza viruses have been shown to induce the expression of TNF-α [47]. TNF-α is an endogenous pyrogen that is involved in a number of acute reactions such as fever, apoptosis, cachexia, inflammation, and inhibition of viral replication [47, 48]. This cytokine may activate NF-κB through TNF receptors (TNFR1 & 2), which mediate the transcription of a vast variety of proteins involved in cell survival, inflammatory reactions, and even those acting against apoptosis [48]. Thus, the compound Q3R could interrupt the effect of the virus on the cytokines which could decrease pro-inflammatory cytokine and increase anti-inflammatory cytokine levels. Evaluation of these cytokine proteins accorded with the genome level results.

## Conclusions

This is the first report of quercetin-3-O-α-L-rhamnopyranoside isolation from *Rapanea melanophloeos* and its immunomodulatory activity against inflammatory reactions of influenza infection. It is suggested that the compound significantly blocked viral particle receptors and prevented cell penetration with reduced viral particle propagation. Pre- and post-penetration treatments did not cause significant changes. This leads to the assumption that the compound does not influence host cellular receptors. Its effect on cytokine expression is a unique highlight for the regulation of inflammatory responses.

Our results described here suggest that Q3R has antiviral activity against influenza A virus and that it may serve as a useful alternative antiviral agent against viral load. In vitro evaluation of the consequences of Q3R showed that this natural compound has the potential to modulate the inflammatory response and efficiently improve the outcome of the influenza disease. This compound could indirectly inhibit the virus, and correspondingly showed the ability to modulate the severity of the disease by changing the cytokine pattern. Further in vivo evaluation is recommended to assist in understanding the benefits of Q3R against influenza disease. The efficacy of this natural compound on cytokine reactions indicates the possible applications of Q3R against a variety of other diseases including infectious or autoimmune disorders. Consequently, quercetin has good potential to decrease the severity of the influenza disease by regulating the innate inflammatory reaction.

## Additional files

Additional file 1: **Figure S1.** ^1^H NMR spectrum of quercetin-3-O-α-L-rhamnopyranoside (in DMSO-d6) (TIF 53 kb)

Additional file 2: **Figure S2.** ^13^C NMR spectrum of quercetin-3-O-α-L-rhamnopyranoside (in DMSO-d6) (TIF 74 kb)

Additional file 3: **Figure S3.** HSQC spectrum of quercetin-3-O-α-L-rhamnopyranoside (in DMSO-d6) (TIF 44 kb)

Additional file 4: **Figure S4.** HMBC spectrum of quercetin-3-O-α-L-rhamnopyranoside (in DMSO-d6) (TIF 48 kb)

Additional file 5: **Figure S5.** H:H COSY spectrum of quercetin-3-O-α-L-rhamnopyranoside (in DMSO-d6) (TIF 86 kb)

## Abbreviations

ANOVA: Analysis of variance
CC_50_: 50% cytotoxic concentration
CCID50: Cell culture infectious dose 50
cRBCs: Chicken red blood cells
DMEM: Dulbecco’s Modified Eagle’s Medium
DMSO: Dimethylsulfoxide
ELISA: Enzyme linked immunosorbant assay
FBS: Fetal Bovine Serum
HA: Hemagglutination assay
IAV: Influenza A virus
M2: Matrix protein 2
MDCK: Madin Darby Canine Kidney
MTT: 3-(4,5-dimethylthiazol-2-yl)-2,5-diphenyltetrazolium bromide
NCTC: Non cytotoxic concentration
NP: Nucleoprotein
OD: Optical Density
PBS: Phosphate-buffered saline
qPCR: Quantitative PCR
RM: *Rapanea melanophloeos*
TLC: Thin Layer Chromatography
Trypsin-TPCK: Tosylamide Phenylethyl Chloromethyl Ketone-treated Trypsin

## References

[1] Webster R, Bean WGO, Chambers T, Kawaoka Y (1992). Evolution and ecology of influenza a viruses. Microbiol Rev.

[2] Fedson DS (2008). Confronting an influenza pandemic with inexpensive generic agents: can it be done?. Lancet Infect Dis.

[3] Pathumwadee I, Chittima L, Thanyada R, Arthorn L, Maturos M, Panita D (2008). How amantadine and rimantadine inhibit proton transport in the M2 protein channel. J Mol Graph Model.

[4] Fiore AE, Shay DK, Broder K, Iskander JK, Tm U, Mootrey G (2008). Prevention and control of influenza: recommendations of the advisory committee on immunization practices. Recomm Rep.

[5] Osterholm MT (2005). Preparing for the next pandemic. N Engl J Med.

[6] Khalafalla MM, Abdellatef E, Dafalla HM, Nassrallah AA, Aboul-Enein KM, Lightfoot DA (2010). Active principle from moringa oleifera lam leaves effective against two leukemias and a hepatocarcinoma. Afr J Biotechnol.

[7] Mozaffari Nejad AS, Kamkar A, Giri A, Pourmahmoudi AA (2013). Ethnobotany and folk medicinal uses of major trees and shrubs in northern Iran. J Med Plants Res.

[8] Amic D, Amie DD, Beslo D, Trinajstic N (2003). Structural-radical scavenging activity relationship of flavonoids. Croatia Chemica Acta.

[9] Aqil F, Ahmad I, Mehmood Z (2006). Antioxidant and free radical scavenging properties of twelve traditionally used Indian medicinal plants. Turk J Biol.

[10] Jalali H, Mozaffari Nejad AS, Ebadi AG, Laey G (2009). Ethnobotany and Folk pharmaceutical properties of major trees or shrubs in northeast of Iran. Asian J Chem.

[11] Street RA, Prinsloo G (2013). Commercially important medicinal plants of South Africa: a review. Journal of Chemistry.

[12] Ruffa MJ, Wagner ML, Suriano M, Vicente C, Nadinic J, Pampuro S (2004). Inhibitory effect of medicinal herbs against RNA and DNA viruses. Antivir Chem Chemother.

[13] Choi HJ (2016). Evaluation of antiviral activity of zanthoxylum species against picornaviruses. Osong Public Health Res Perspect.

[14] Hwang BS, Lee IK, Choi HJ, Yun BS (2015). Anti-influenza activities of polyphenols from the medicinal mushroom Phellinus baumii. Bioorg Med Chem Lett.

[15] Zink T, Chaffin J (1998). Herbal health products: what family physicians need to know. Am Fam Physician.

[16] Milugo TK, Omosa LK, Ochanda JO, Owuor BO, Wamunyokoli FA, Oyugi JO (2013). Antagonistic effect of alkaloids and saponins on bioactivity in the quinine tree (Rauvolfia caffra sond.): further evidence to support biotechnology in traditional medicinal plants. BMC Complement Altern Med.

[17] Gwala PE (2011). Anti-platelet aggregation activity of Rapanea melanophloeos -A Zulu medicinal plant, in Department of Biochemistry and Microbioogy.

[18] Mehrbod P, Abdalla MA, Njoya EM, Ahmed AS, Fotouhi F, Farahmand B (2018). South African medicinal plant extracts active against influenza A virus. BMC Complement Altern Med.

[19] Karber G (1931). 50% endpoint calculation archive for experimental pathology and. Pharmacology.

[20] Mehrbod P, Ideris A, Omar AR, Hair-Bejo M (2012). Evaluation of antiviral effect of atorvastatin on H1N1 infection in MDCK cells. Afr J Microbiol Res.

[21] Mosmann T (1983). Rapid colorimetric assay for cellular growth and survival: application to proliferation and cytotoxicity assays. J Immunol Methods.

[22] Mehrbod P, Motamed N, Tabatabaian M, Soleimani-Estyar R, Amini E, Shahidi M (2009). In vitro antiviral effect of “Nanosilver” on influenza virus. Daru.

[23] Hirst GK (1942). The quantitative determination of influenza virus and antibodies by means of red cell agglutination. J Exp Med.

[24] Godornes C, Leader BT, Molini BJ, Centurion-Lara A, Lukehart SA (2007). Quantitation of rabbit cytokine mRNA by real-time RT-PCR. Cytokine.

[25] Chapman, Hall. Chapman-hall dictionary of natural products on CD-ROM, Chemical DataBase (2017).

[26] Chattopadhyay D, Sarkar MC, Chatterjee T, Sharma Dey R, Bag P, Chakraborti S (2009). Recent advancements for the evaluation of anti-viral activities of natural products. New Biotechnol.

[27] Kobasa D, Jones SM, Shinya K, Kash JC, Copps J, Ebihara H (2007). Aberrant innate immune response in lethal infection of macaques with the 1918 influenza virus. Nature.

[28] Piqueras B, Connolly J, Freitas H, Palucka AK, Banchereau J (2006). Upon viral exposure mye-loid and plasmacytoid dendritic cells produce three waves of distinct chemokines to recruitimmune effectors. Blood.

[29] Liang-Tzung L, Wen-Chan H, Chun-Ching L (2014). Antiviral natural products and herbal medicines. J Tradit Complement Med.

[30] Kumar S, Abhay KP (2013). Chemistry and biological activities of flavonoids: an overview. Sci World J.

[31] Kaul TN, Middleton E, Ogra PL (1985). Antiviral effect of flavonoids on human viruses. J Med Virol.

[32] Orhan DD, Özçelik B, Özgen S, Ergun F (2010). Antibacterial, antifungal, and antiviral activities of some flavonoids. Microbiol Res.

[33] Zandi K, Teoh BT, Sam SS, Wong PF, Mustafa MR, AbuBakar S (2011). Antiviral activity of four types of bioflavonoid against dengue virus type-2. Virol J.

[34] Hafidh RR, Abdulamir AS, Jahanshiri F, Abas F, Abu Bakar F, Sekawi Z (2009). Asia is the mine of natural antiviral products for public health. Open Complement Med J.

[35] Dayem AA, Choi HY, Kim YB, Cho SG (2015). Antiviral effect of methylated flavonol isorhamnetin against influenza. PLoS One.

[36] Hossain MK, Choi HY, Hwang JS, Dayem AA, Kim JH, Kim YB (2014). Antiviral activity of 3,4′-dihydroxyflavone on influenza a virus. J Microbiol.

[37] Arena A, Bisignano G, Pavone B, Tomaino A, Bonina FP, Saija A (2008). Antiviral and immunomodulatory effect of a lyophilized extract of Capparis spinosa L. buds. Phytother Res.

[38] Chiang LC, Ng LT, Chiang W, Chang MY, Lin CC (2003). Immunomodulatory activities of flavonoids, monoterpenoids, triterpenoids, iridoid glycosides and phenolic compounds of Plantago species. Planta Med.

[39] Aichour R, Charef N, Baghiani A, Arrar L (2016). Immunomodulatory effects of Algerian caper. Int J Pharm Pharm Sci.

[40] Miles SL, McFarland M, Niles RM (2014). Molecular and physiological actions of quercetin: need for clinical trials to assess its benefits in human disease. Nutr Rev.

[41] Wu W, Li R, Li X, He J, Jiang S, Liu S (2016). Quercetin as an antiviral agent inhibits influenza a virus (IAV) entry. Viruses.

[42] Choi HJ, Song JH, Park KS, Kwon DH (2009). Inhibitory effects of quercetin 3-rhamnoside on influenza a virus replication. Eur J Pharm Sci.

[43] Gannagé M, Dormann D, Albrecht R, Dengjel J, Torossi T, Rämer PC (2009). Matrix protein 2 of influenza a virus blocks autophagosome fusion with lysosomes. Cell Host Microbe.

[44] Nayak DP, Hui EKW, Barman S (2004). Assembly and budding of influenza virus. Virus Res.

[45] Bouvier NM, Palese P (2008). The biology of influenza viruses. Vaccine.

[46] Sun J, Dodd H, Moser EK, Sharma R, Braciale TJ (2011). CD4+ T cell help and innate-derived IL-27 induce Blimp-1-dependent IL-10 production by antiviral CTLs. Nat Immunol.

[47] Cheung C, Poon L, Lau A, Luk W, Lau Y, Shortridge K (2002). Induction of proinflammatory cytokines in human macrophages by influenza a (H5N1) viruses: a mechanism for the unusual severity of human disease?. Lancet.

[48] Swardfager W, Lanctôt K, Rothenburg L, Wong A, Cappell J, Herrmann N (2010). Tumor necrosis factor alpha. Biol Psychiatry.

