# Supplementary figures and images for: Immunomodulatory properties of quercetin-3-O-α-L-rhamnopyranoside from Rapanea melanophloeos against influenza a virus
Source: BMC Complement Altern Med. 2018 Jun 15;18:184. doi: 10.1186/s12906-018-2246-1 (PMC6003079; doi:10.1186/s12906-018-2246-1)

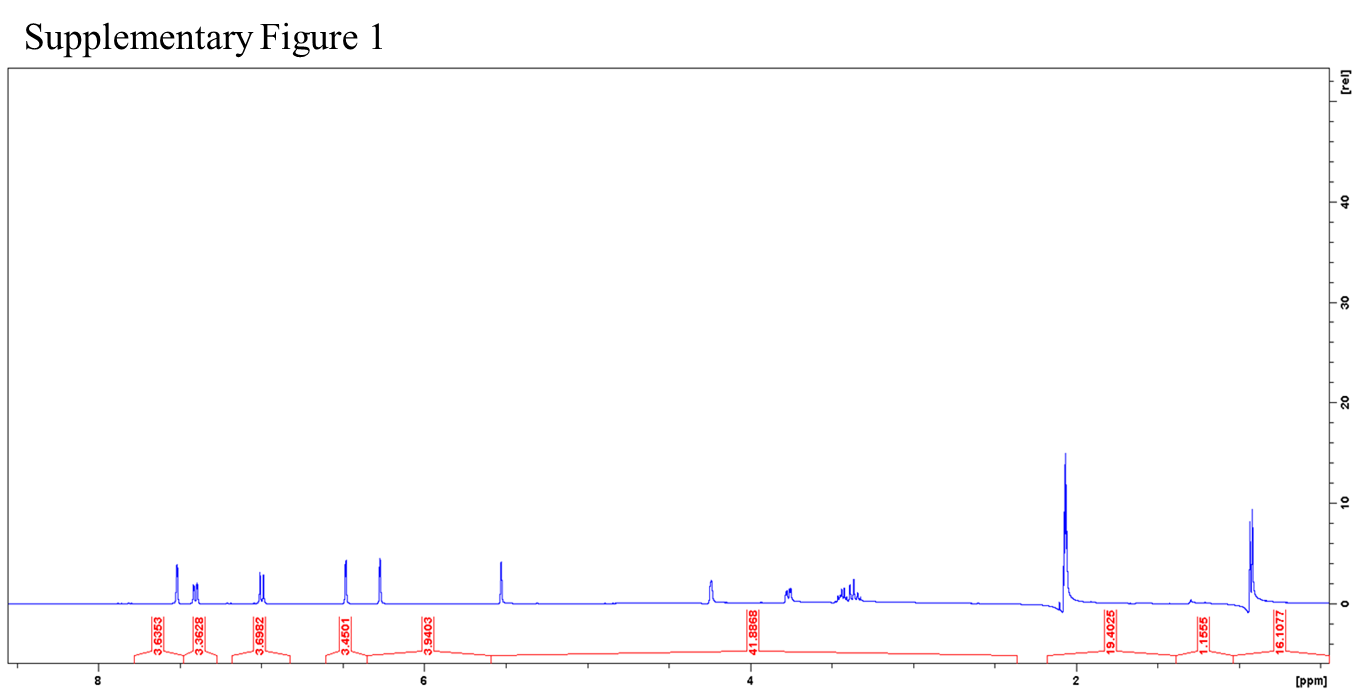

Supplement: Supplementary file 1 — Figure S1. 1H NMR spectrum of quercetin-3-O-α-L-rhamnopyranoside (in DMSO-d6) (TIF 53 kb) [file 12906_2018_2246_MOESM1_ESM.tif]

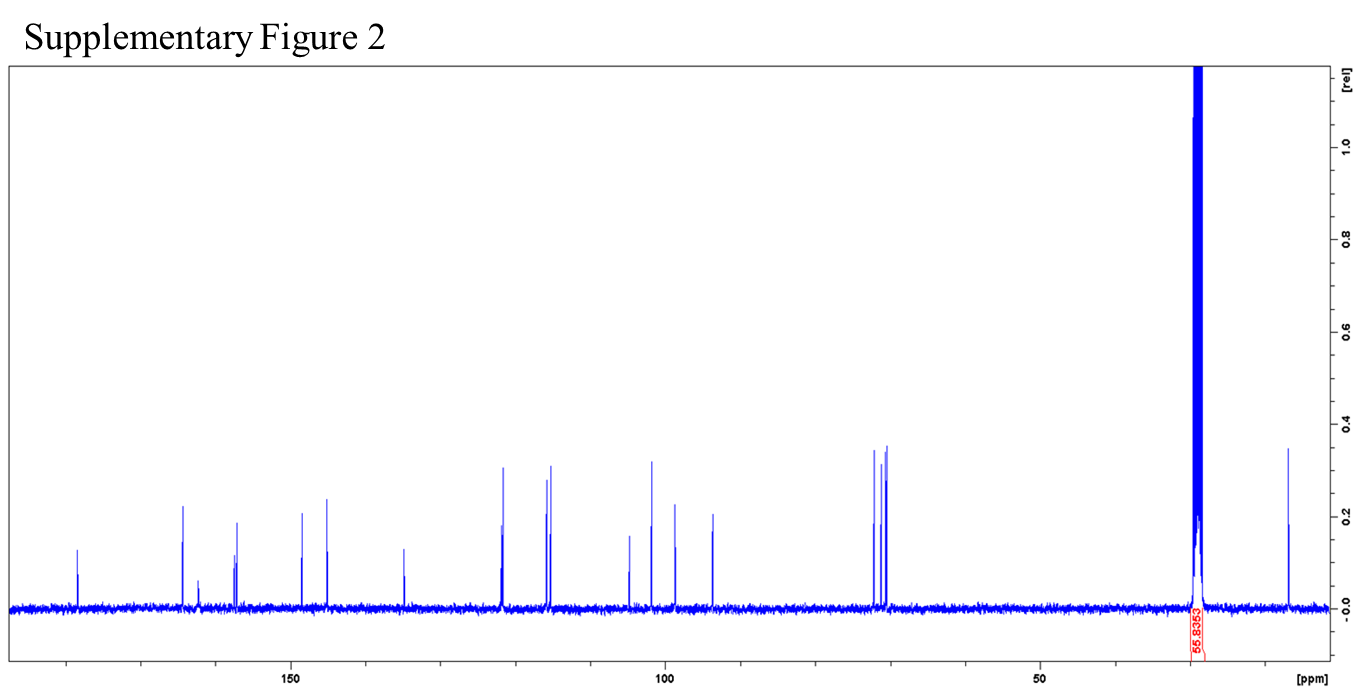

Supplement: Supplementary file 2 — Figure S2. 13C NMR spectrum of quercetin-3-O-α-L-rhamnopyranoside (in DMSO-d6) (TIF 74 kb) [file 12906_2018_2246_MOESM2_ESM.tif]

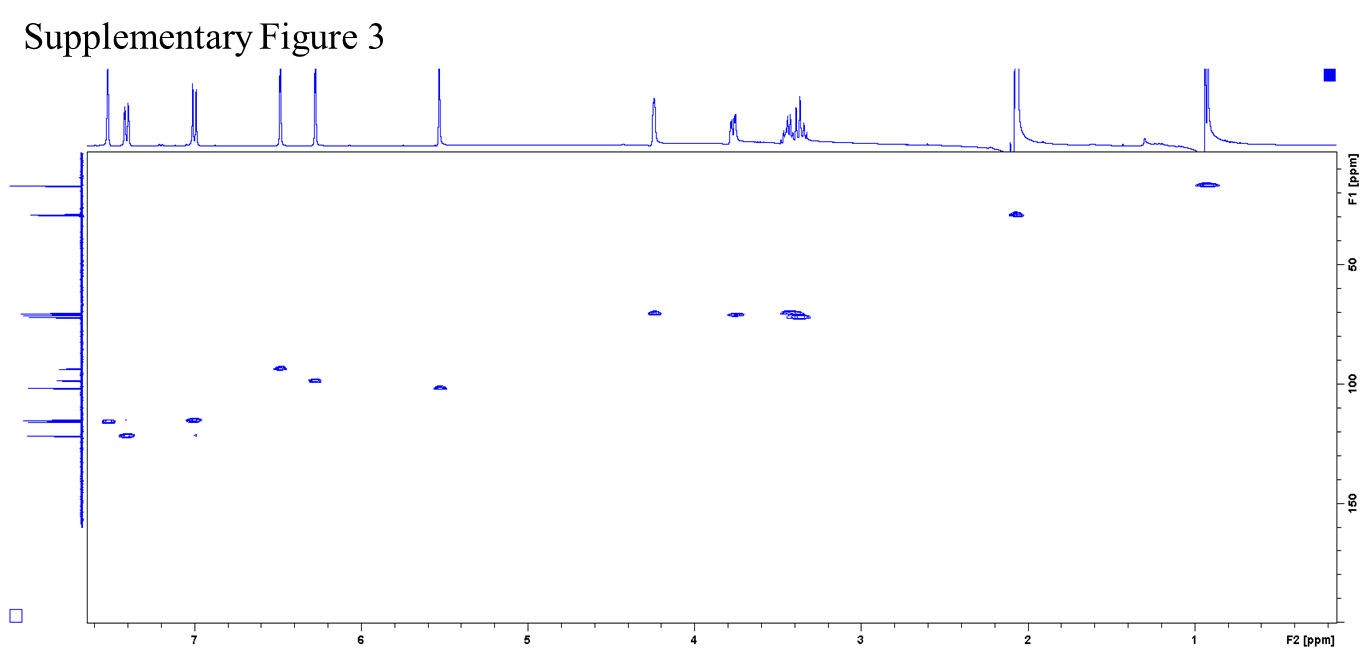

Supplement: Supplementary file 3 — Figure S3. HSQC spectrum of quercetin-3-O-α-L-rhamnopyranoside (in DMSO-d6) (TIF 44 kb) [file 12906_2018_2246_MOESM3_ESM.tif]

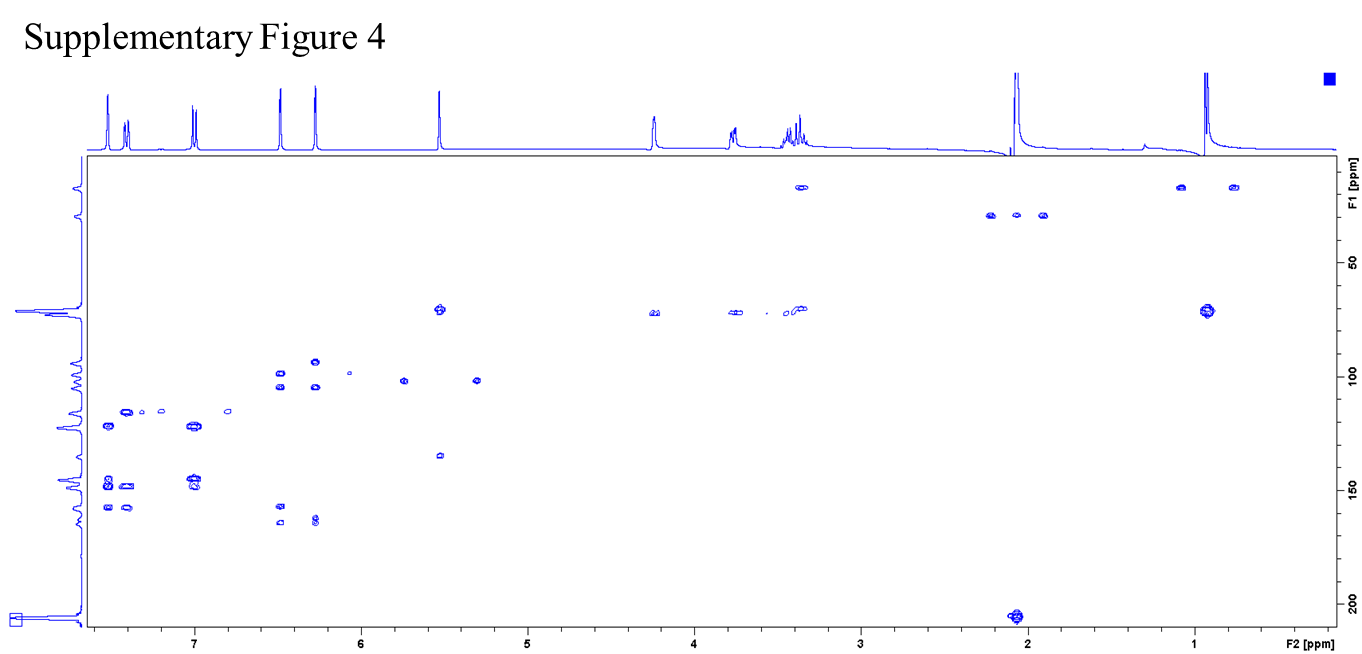

Supplement: Supplementary file 4 — Figure S4. HMBC spectrum of quercetin-3-O-α-L-rhamnopyranoside (in DMSO-d6) (TIF 48 kb) [file 12906_2018_2246_MOESM4_ESM.tif]

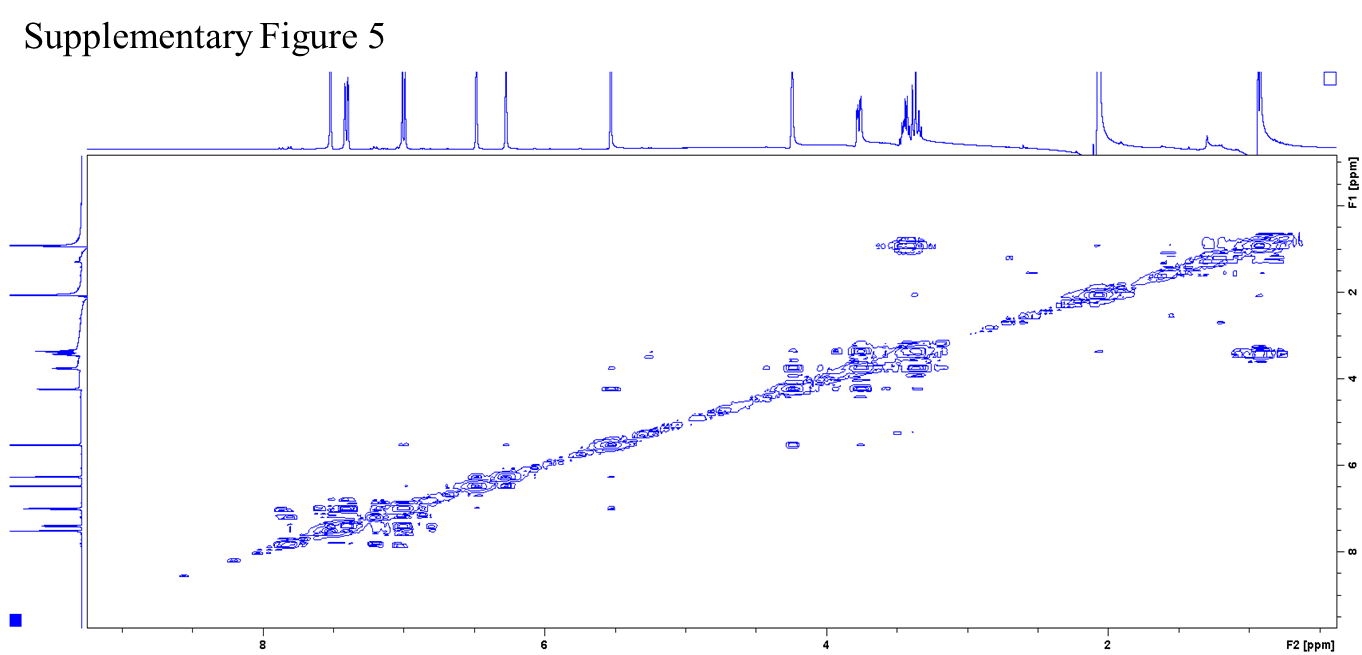

Supplement: Supplementary file 5 — Figure S5. H:H COSY spectrum of quercetin-3-O-α-L-rhamnopyranoside (in DMSO-d6) (TIF 86 kb) [file 12906_2018_2246_MOESM5_ESM.tif]
